# The prevention indicator system of the German Federal States – a tool to monitor prevention policies

**DOI:** 10.1093/eurpub/ckac129.759

**Published:** 2022-10-25

**Authors:** JD Finger, S Oberwoehrmann, J Zeiher, M Haftenberger, S Hermann

**Affiliations:** Department of Health, Senate Department for Health, Berlin, Germany

## Abstract

In 2015 the German Prevention Act was implemented. The National Prevention Conference published the first National Prevention Report in 2019 to evaluate the health promotion activities. The second National Prevention Report is planned for 2023. Development of a harmonized prevention reporting system for the German Federal States is needed to form the basis for the contribution of the Federal States to the next National Prevention Report. A working group mandated from the sub-national health authorities has developed a harmonized prevention reporting system for the German Federal States since 2018. The Robert Koch Institute collaborated as representative of the national level during the process. Subject areas for indicators were selected based on a survey in which all 16 State Ministries of Health participated. Indicator subgroups developed indicators for each subject area based on predefined indicator selection criteria. Final set of indicators was adopted by indicator rating and majority voting process. The German Health Ministers Conference acknowledged the indicator system in June 2021. The conceptual framework is adapted from the health determinants model of Dahlgren and Whitehead. The indicator system is divided into 14 subject areas categorized into upstream, midstream and downstream level of prevention indicators. Seventy-three prevention indicators were included as a whole. The indicator short list consists of 32 Core indicators. An overview of the prevention indicator system will be given. First results of a pilot data collection will be shown. Health promotion and prevention reporting tools are needed to monitor prevention policies and evaluate health promotion measures. The prevention indicator system of the German Federal States will be used for the National Prevention Strategy in Germany of which one component is the next National Prevention Report 2023.

**Key messages:**

• The prevention indicator system of the German Federal States is a useful tool to monitor prevention policies.

• The indicator system will form the basis for the German Federal States’ contribution to the National Prevention Report 2023.

